# The human glomerular endothelial cells are potent pro-inflammatory contributors in an *in vitro* model of lupus nephritis

**DOI:** 10.1038/s41598-019-44868-y

**Published:** 2019-06-06

**Authors:** Paraskevi Dimou, Rachael D. Wright, Kelly L. Budge, Angela Midgley, Simon C. Satchell, Matthew Peak, Michael W. Beresford

**Affiliations:** 1 Department of Women’s and Children’s Health, Institute of Translational Medicine, University of Liverpool, Liverpool, UK; 2 NIHR Alder Hey Clinical Research Facility, Alder Hey Children’s NHS Foundation Trust, Liverpool, UK; 3 Academic Renal Unit, University of Bristol, Bristol, UK; 4 Department of Paediatric Rheumatology, Alder Hey Children’s NHS Foundation Trust, Liverpool, UK

**Keywords:** Autoimmunity, Lupus nephritis

## Abstract

Juvenile-onset lupus nephritis (LN) affects up to 80% of juvenile-onset systemic lupus erythematosus patients (JSLE). As the exact role of human renal glomerular endothelial cells (GEnCs) in LN has not been fully elucidated, the aim of this study was to investigate their involvement in LN. Conditionally immortalised human GEnCs (ciGEnCs) were treated with pro-inflammatory cytokines known to be involved in LN pathogenesis and also with LPS. Secretion and surface expression of pro-inflammatory proteins was quantified via ELISA and flow cytometry. NF-κΒ and STAT-1 activation was investigated via immunofluorescence. Serum samples from JSLE patients and from healthy controls were used to treat ciGEnCs to determine via qRT-PCR potential changes in the mRNA levels of pro-inflammatory genes. Our results identified TNF-α, IL-1β, IL-13, IFN-γ and LPS as robust *in vitro* stimuli of ciGEnCs. Each of them led to significantly increased production of different pro-inflammatory proteins, including; IL-6, IL-10, MCP-1, sVCAM-1, MIP-1α, IP-10, GM-CSF, M-CSF, TNF-α, IFN-γ, VCAM-1, ICAM-1, PD-L1 and ICOS-L. TNF-α and IL-1β were shown to activate NF-κB, whilst IFN-γ activated STAT-1. JSLE patient serum promoted IL-6 and IL-1β mRNA expression. In conclusion, our *in vitro* model provides evidence that human GEnCs play a pivotal role in LN-associated inflammatory process.

## Introduction

Lupus nephritis (LN) is one of the main complications of juvenile-onset systemic lupus erythematosus (JSLE), a rare, multi-system autoimmune disease^1^. LN is a chronic inflammatory renal disease characterised by periodic flares (active LN) and remissions (inactive LN)^2^. Each flare potentially contributes to the accumulation of damage of renal structures^3^ such as the glomerulus^4^. LN can affect up to 80% of JSLE patients^5–7^, whereas 10–15% of LN patients eventually develop end-stage renal disease^8–12^.

The renal glomerulus is part of the nephron, where primary urine formation occurs^13^. Each glomerulus contains a network of glomerular capillaries which, in healthy individuals, effectively filter blood plasma through the glomerular filtration barrier (GFB) restricting blood cells and proteins inside the circulation^13^. In LN, chronic inflammation-induced GFB damage results in proteinuria and haematuria^14,15^.

The human glomerular endothelial cells (GEnCs), a component of the GFB, line the glomerular capillary lumen^13^. As the GEnCs directly interact with circulating factors and immune cells in the blood and with the renal glomerular podocytes and mesangial cells, they could be central intra-renal contributors to inflammatory processes. To date, there are limited data concerning human GEnC involvement in LN inflammation and production of pro-inflammatory mediators. However, human umbilical vein endothelial cells (HUVECs)^16,17^ and human brain microvascular endothelial cells (HBMECs)^18,19^, when cultured *in vitro* under appropriate pro-inflammatory stimuli such as tumour necrosis factor-alpha (TNF-α), interleukin (IL)-1 beta (IL-1β) and lipopolysaccharide (LPS), have been shown to elicit inflammatory responses. These responses involve nuclear factor kappa-light-chain-enhancer of activated B-cells (NF-κB) activation and production of adhesion molecules and cytokines. Thus, the aim of this study was to investigate the potential inflammatory role of human GEnCs in an *in vitro* model of LN. Unravelling their potential involvement in lupus renal disease could provide further insight into LN pathogenesis.

## Results

### Inflammatory stimulation of ciGEnCs induces the production of urinary biomarkers and pro-inflammatory proteins

**Figure 1:**
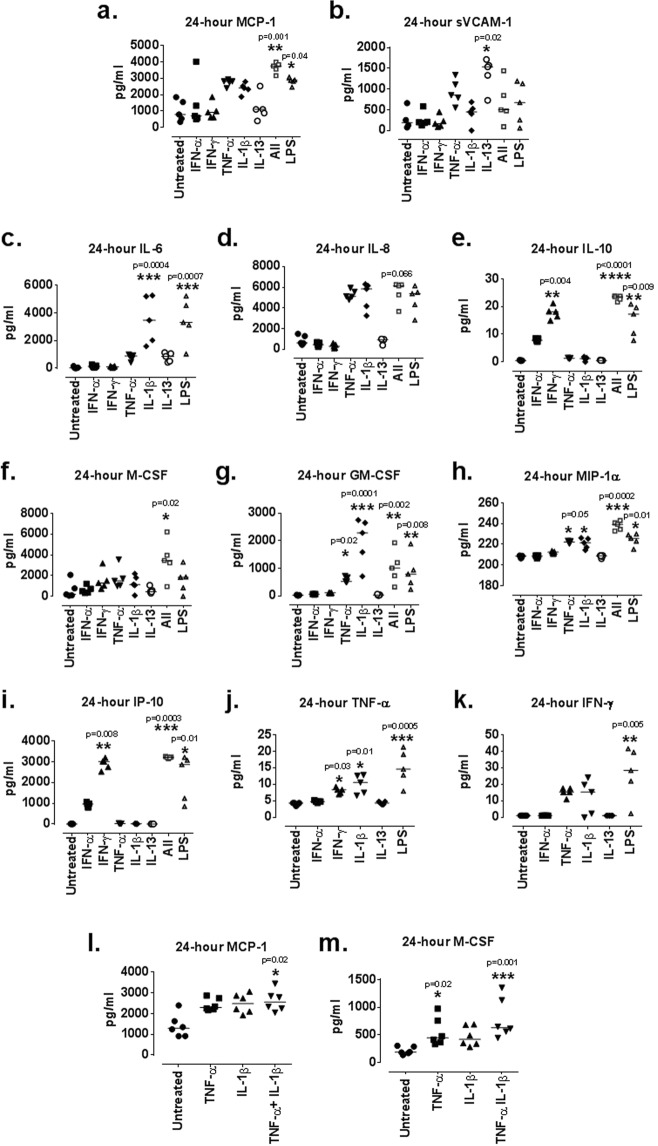
LN urinary biomarker, chemokine, cytokine and growth factor secretion after 24-hour ciGEnC-stimulation with individual cytokines, combination of all cytokines (All) and LPS. MCP-1 (**a**), sVCAM-1 (**b**), IL-6 (**c**), IL-8 (**d**), IL-10 (**e**), M-CSF (**f**), GM-CSF (**g**), MIP-1α (**h**), IP-10 (**i**), TNF-α (**j**) and IFN-γ (**k**) secretion in response to cytokine treatments. MCP-1 (l) and M-CSF secretion (m) following combined TNF-α and IL-1β treatments. N = 5–6/group, Data are presented as median concentrations (pg/ml) [range] are analysed using Kruskal-Wallis test with Dunn’s post-hoc test, *P < 0.05, **P < 0.01, ***P < 0.001, ****P < 0.0001 vs untreated.

The combination of all cytokines (All: 3,755 pg/mL [3,159–3,989], p = 0.008) led to statistically significantly higher levels of secreted monocyte chemoattractant protein-1 (MCP-1) compared to the untreated cells (800 pg/mL [346–1,856]) (Fig. 1a), an effect also observed for LPS (2,876 pg/mL [2,457–3,072], p = 0.04). A significant increase in secreted levels of soluble vascular cell adhesion molecule-1 (sVCAM-1) (Fig. 1b) was induced only by IL-13 (1,539 pg/mL [731–1,718], p = 0.022) compared to the untreated cells (192 pg/ml [74–661]). None of the other novel urinary biomarkers were expressed by ciGEnCs (data not shown).

As the ciGEnCs were shown to mainly produce and secrete only two (MCP-1 and sVCAM-1) out of the seven identified novel urinary biomarkers for LN^20^, it was hypothesised that the role of GEnCs may be related to the production and local secretion of other pro-inflammatory mediators. To address this hypothesis, changes in secretion levels of pro-inflammatory cytokines (TNF-α, IFN-γ, IL-6 and IL-10), chemokines (macrophage inflammatory protein-1 alpha (MIP-1α), IFN-γ-induced protein-10 (IP-10) and IL-8) as well as the blood cell growth factors (granulocyte-macrophage and macrophage colony-stimulating factor (GM-CSF and M-CSF)) were examined via Luminex ELISA.

IL-6 secretion was significantly upregulated after IL-1β (3,479 pg/mL [1,599–5,273], p = 0.0004) and LPS stimulation (3,323 pg/mL [1,083–5,228], p = 0.0007) (Fig. 1c) compared to untreated ciGEnCs (31 pg/ml [11–164]). A trend was observed for increased IL-8 secretion after combined cytokine treatment (All; 6,127 pg/mL [3,664–6,255], p = 0.066) compared to untreated cells (623 pg/mL [449–1,503]) (Fig. 1d). Combined cytokine treatment (All: 24 pg/ml [22–24], p < 0.0001) and LPS (17 pg/ml [8–21], p = 0.009) significantly increased IL-10 secretion compared to untreated cells (0.5 pg/ml [0.3–0.7]) (Fig. 1e). The combined cytokine treatment led to secretion of significantly higher amounts of M-CSF (All: 3,461 pg/ml [946–6,220], p = 0.02) compared to untreated cells (192 pg/ml [69–2,077]) (Fig. 1f). IL-1β (2,284 pg/ml [710–2,748], p = 0.0001) and TNF-α (522 pg/ml [493–722], p = 0.02) significantly increased the secretion of GM-CSF compared to untreated cells (20 pg/ml [17–40]), as did the combined cytokine treatment (All: 1,010 pg/ml [323–1,925], p = 0.002) and LPS (790 pg/ml [231–1,882], p = 0.008) (Fig. 1g).

TNF-α (222.3 pg/ml [221–224], p = 0.048), IL-1β (221.5 pg/ml [214–226], p = 0.049) and the combined cytokine treatment (All: 239 pg/ml [233–243], p = 0.0002) significantly increased the secretion of MIP-1α compared to untreated cells (209 pg/ml [207–209], as did LPS (226 pg/ml [215–230], p = 0.011) (Fig. 1h). IFN-γ (3,015 pg/ml [2,530–3,201], p = 0.008) and combination of cytokines (All: 3,222 pg/ml [3,153–3,262], p = 0.0003) significantly upregulated IP-10 secretion compared to untreated cells (2 pg/ml [2–5]), as did LPS (2,870 pg/ml [847–3,218], p = 0.013) (Fig. 1i).

IFN-γ (8 pg/ml [7–9], p = 0.029), IL-1β (11 pg/ml [7–13], p = 0.011) and LPS (15 pg/ml [8–21], p = 0.0005) treatments significantly upregulated TNF-α secretion compared to untreated cells (4 pg/ml [4–4]) (Fig. 1j). The single TNF-α treatment and the combined cytokine treatment were excluded as they gave false positive results due to the presence of recombinant human TNF-α. LPS treatment (28 pg/ml [2–42], p = 0.005) induced the secretion of significantly higher amounts of IFN-γ compared to untreated cells (1 pg/ml [1–1]) (Fig. 1k). The single IFN-γ treatment and the combined cytokine treatment were excluded as they gave false positive results due to the presence of recombinant IFN-γ.

Due to lack of IL-6- and VEGF-induced changes in the mRNA expression levels of MCP-1, VCAM-1, IL-6 and IL-8 compared to untreated ciGEnCs (data not shown), ciGEnC pro-inflammatory protein secretion in IL-6- and VEGF-treated conditioned media was not tested in ELISA assays (the 96-well plate capacity of the Luminex assay also limited the amount of conditioned media tested to those that were expected to exhibit the most meaningful changes in protein secretion compared to the untreated ciGEnCs). However, IL-6 and VEGF were included in the combined cytokine treatments (All).

The combined effect of TNF-α and IL-1β in MCP-1 and M-CSF secretion was also specifically tested. TNF-α together with IL-1β statistically significantly increased MCP-1 secretion (2,561 pg/ml [2,055–3,449], p = 0.02) compared to untreated ciGEnCs (1,300 pg/ml [916–2,401]) (Fig. 1l). TNF-α alone significantly increased M-CSF secretion (446 pg/ml [333–975], p = 0.02) compared to untreated ciGEnCs (187 pg/ml [136–304]) and combined TNF-α and IL-1β treatment further increased M-CSF secretion (629 pg/ml [446–1,354], p = 0.001) (Fig. 1m).

### 24-hour serum treatments induce changes in mRNA expression levels of pro-inflammatory genes

**Figure 2:**
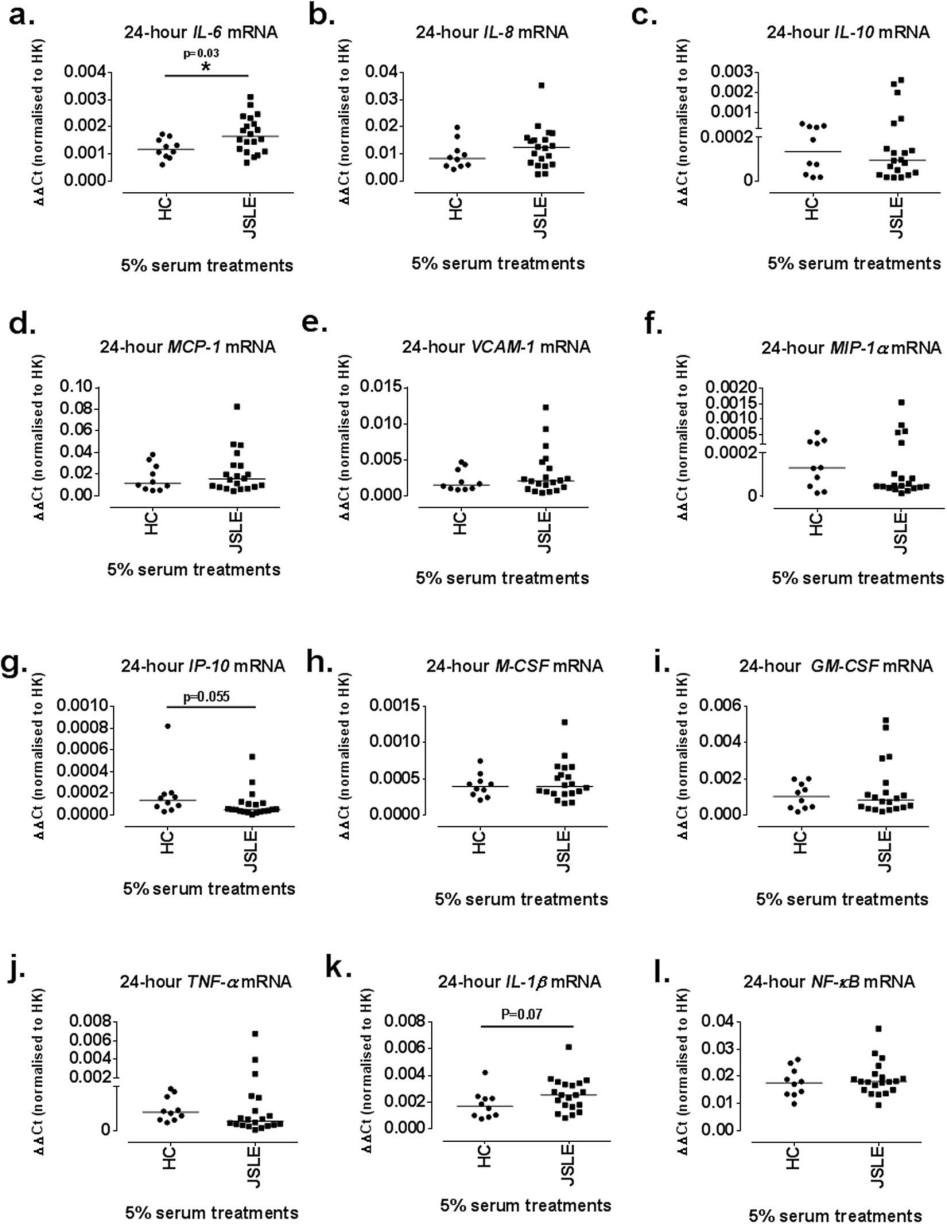
Effect of 24 h ciGEnC treatments with 5% JSLE or HC sera in pro-inflammatory gene mRNA expression. Changes in *IL-6* (**a**), *IL-8* (**b**), *IL-10* (**c**), *M-CSF* (**d**), *GM-CSF* (**e**), *MCP-1* (**f**), *VCAM-1* (**g**), *MIP-1α* (**h**), *IP-10* (**i**), *TNF-α* (**j**), *IL-1β* (**k**) and *NF-κB* (**l**) mRNA expression levels. Data presented as median ΔΔCt [range] are analysed with Mann-Whitney test. *P </= 0.05 vs HC N = 10–20/group.

Treatment of ciGEnCs for 4 h with sera from JSLE- and healthy control (HC) patients did not show any significant differences between groups (data not shown). At 24 h, JSLE serum (0.002 [0.0007–0.003], p = 0.03) significantly increased *IL-6* mRNA expression compared to HC (0.001 [0.0006–0.002]) (Fig. 2a). A trend was also observed for increased JSLE sera-induced *IL-1β* mRNA expression (0.003 [0.0008–0.006], p = 0.07) compared to HC (0.002 [0.0008–0.004]) (Fig. 2k). Furthermore, a trend was observed for reduced *IP-10* mRNA expression levels after JSLE serum treatments (0.00005 [0.000004–0.005], p = 0.055) compared to HC sera (0.0001 [0.00003–0.00080]) with the mRNA in both groups being, in general, lowly expressed (Fig. 2g).

**Figure 3:**
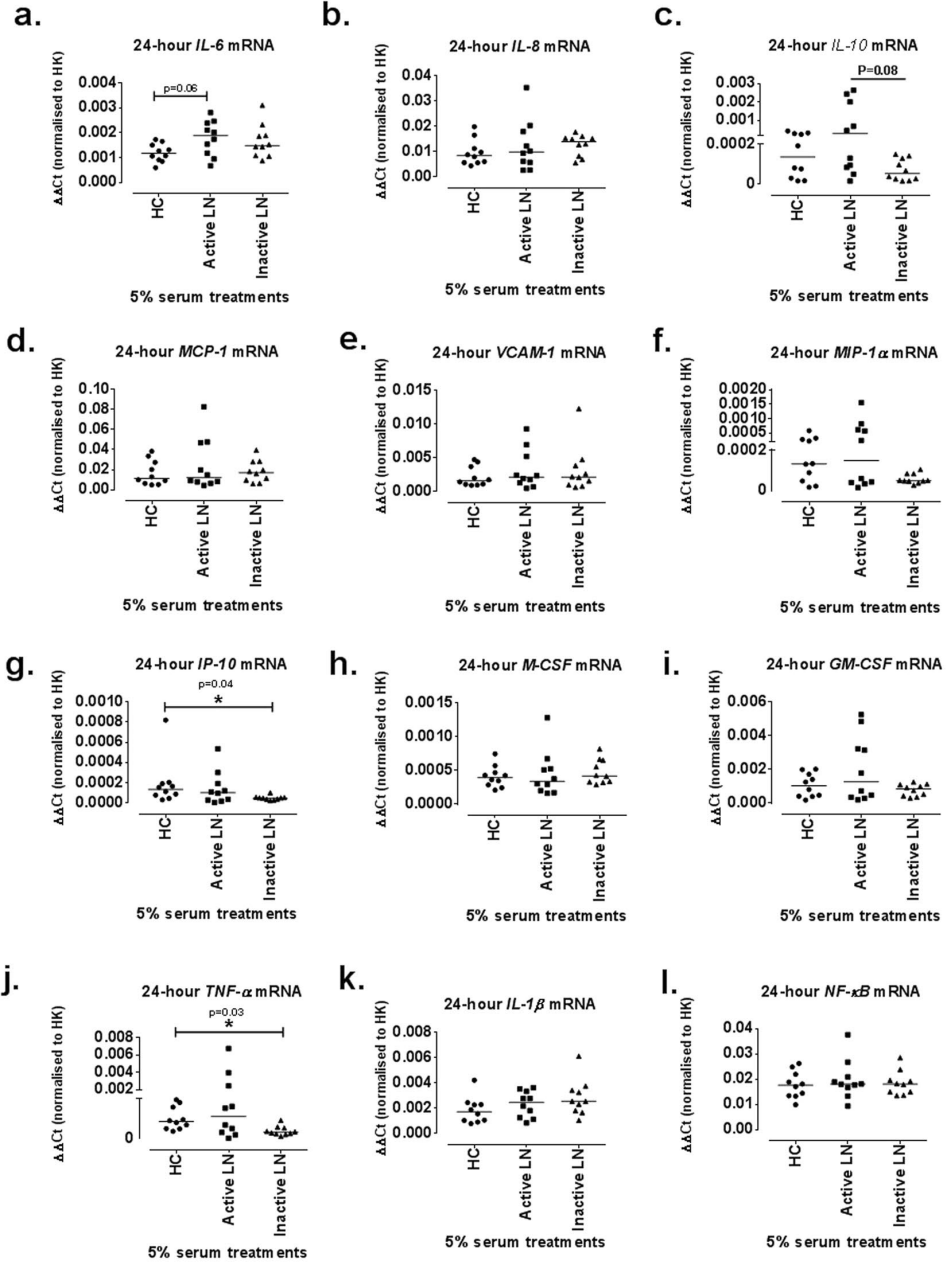
Effect of 24 h ciGEnC treatments with 5% active and inactive LN or HC sera in pro-inflammatory gene mRNA expression. Changes in *IL-6* (**a**), *IL-8* (**b**), *IL-10* (**c**) *M-CSF* (**d**), *GM-CSF* (**e**), *MCP-1* (**f**), *VCAM-1* (**g**), *MIP-1α* (**h**),* IP-10* (**i**), *TNF-α* (**j**), *IL-1β* (**k**) and *NF-κB* (**l**) mRNA expression levels. Data presented as median ΔΔCt [range] are analysed with Kruskal-Wallis with Dunn’s post-hoc test. *P </= 0.05. N = 10/group.

When JSLE serum was categorised as either active (renal BILAG A/B) or inactive LN (renal BILAG D/E), a trend was observed for decreased *IP-10* mRNA levels after inactive LN serum treatments (0.00005 [0.00002–0.0001], p = 0.06) compared to HC (0.0001 [0.00003–0.0008]) but no significant difference was demonstrated between HC and active LN serum treatments (Fig. 3g). Similarly, *TNF-α* mRNA expression levels were statistically significantly reduced after inactive LN serum treatments (0.0003 [0.0001–0.000901], p = 0.04) but not after active LN serum treatments when compared to HC (0.0008 [0.0004–0.002]) (Fig. 3j). A trend was observed for higher *IL-10* mRNA expression after active LN serum treatments (0.0003 [0.00002–0.003]) compared to the inactive sera (0.00005 [0.00002–0.0001]) although these differences were not statistically significant (p = 0.08) (Fig. 3c).

### 24-hour serum treatments induce changes in the secretion levels of IL-6

**Figure 4:**
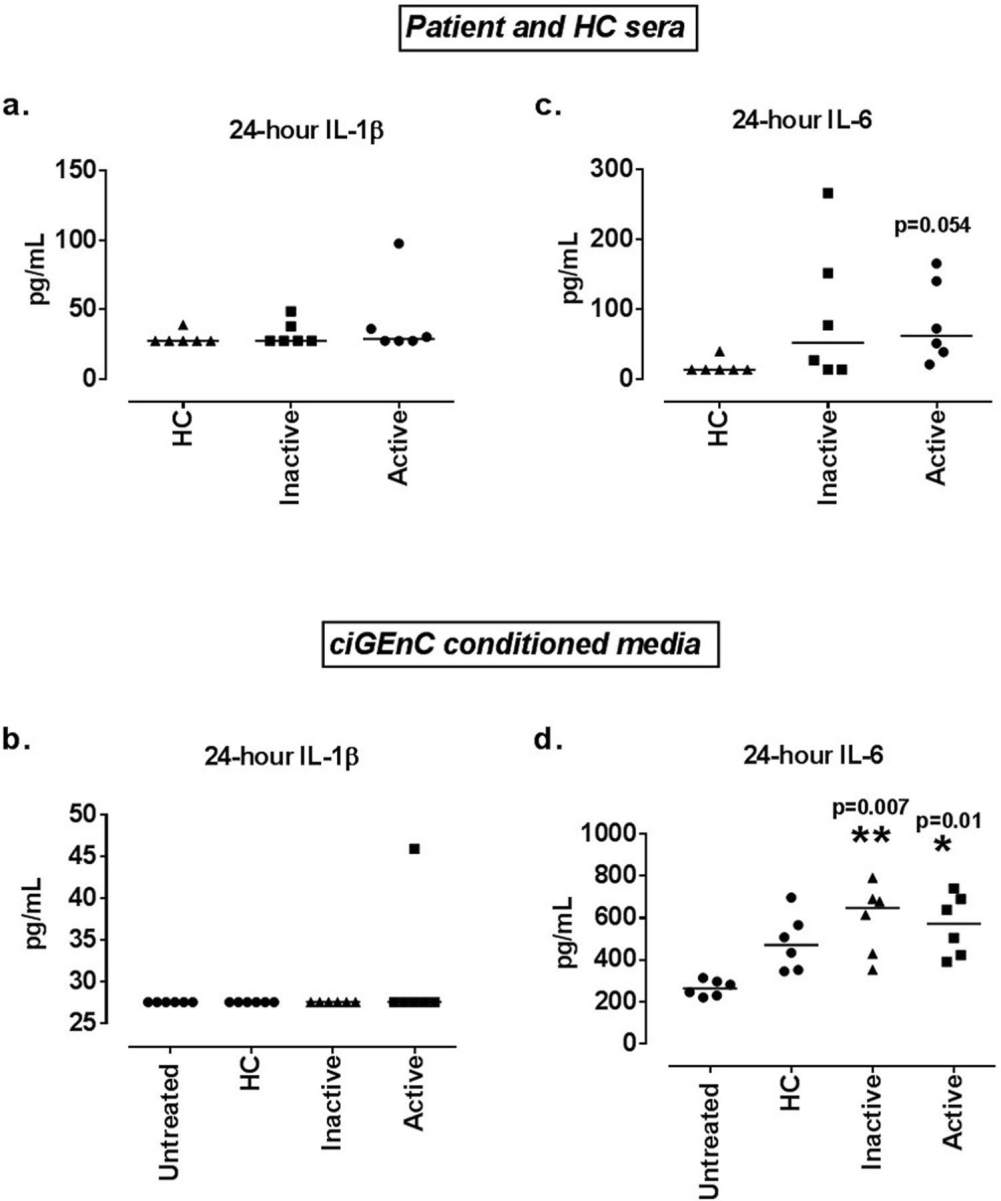
Effect of 24 h ciGEnC treatments with 5% active and inactive LN or HC sera in IL-1β and IL-6 secretion. IL-1β levels in active and inactive LN patient and HC sera **(a)** and IL-1β levels in ciGEnC conditioned media **(b)**. IL-6 levels in active and inactive LN patient and HC sera **(c)** and IL-6 levels in ciGEnC conditioned media **(d)**. Data presented as median concentrations (pg/ml) [range] are analysed using Kruskal-Wallis test with Dunn’s post-hoc test. *P </= 0.05, **P < 0.01 vs HC or vs untreated ciGEnC conditioned media. N = 6/group.

The 24 h JSLE serum treatments were able to promote an increase in the mRNA expression of *IL-1β* and *IL-6*. For this reason, we also investigated the effect of serum treatments in IL-1β and IL-6 secretion by the ciGEnCs. IL-1β and IL-6 in serum samples were also tested to ensure whether potential presence of these cytokines in the ciGEnC conditioned media could be attributed to ciGEnC production. IL-1β presence in serum samples was relatively low (frequently below the level of detection for the assay) and did not differ among HCs, active and inactive LN patients (Fig. 4a). Furthermore, when ciGEnCs were treated with 5% sera from LN patients, IL-1β levels secreted by the cells were below the level of detection for the assay (except for 1 active disease sera sample) (Fig. 4b).

On the contrary, a trend was observed for higher IL-6 levels in active LN sera (62 pg/ml [21–165], p = 0.054) compared to those of HC sera (13 pg/ml [13–40]) whereas inactive LN sera levels of IL-6 (52 pg/ml [1–267]) were closer to those of active LN sera (Fig. 4c). The active LN (572 pg/ml [390–741], p = 0.01]) and the inactive LN (647 pg/ml [352–792], p = 0.007) serum treatments promoted a statistically significant increase in IL-6 secretion by ciGEnCs compared to untreated ciGEnCs (263 pg/ml [22–314]); the HC serum treatments (471 pg/ml [345–698]) were not able induce as high IL-6 levels as the JSLE sera.

### Inflammatory protein release is not associated with increased cellular death

The majority of ciGEnCs remained viable (Anx V−/PI−) after 24 h treatment with cytokines and LPS similarly to the untreated ciGEnCs (Supplementary Fig. 1). No significant changes were observed in the levels of apoptotic (Anx V+/PI−) and late apoptotic/necrotic (Anx V+/PI+) ciGEnCs among the different treatments (Supplementary Fig. 1).

### Inflammatory mediators induce cell adhesion and co-stimulatory molecule expression in ciGEnCs

The endothelium is responsible for the recruitment and activation of leukocytes to the glomerular space by upregulating expression of cellular adhesion and co-stimulatory molecules. The role of TNF-α, IL-1β, IL-13, IFN-γ and LPS was therefore assessed in modulating VCAM-1, intercellular adhesion molecule-1 (ICAM-1), programmed death-ligand 1 (PD-L1) and inducible co-stimulator-ligand (ICOS-L) surface expression.

**Figure 5:**
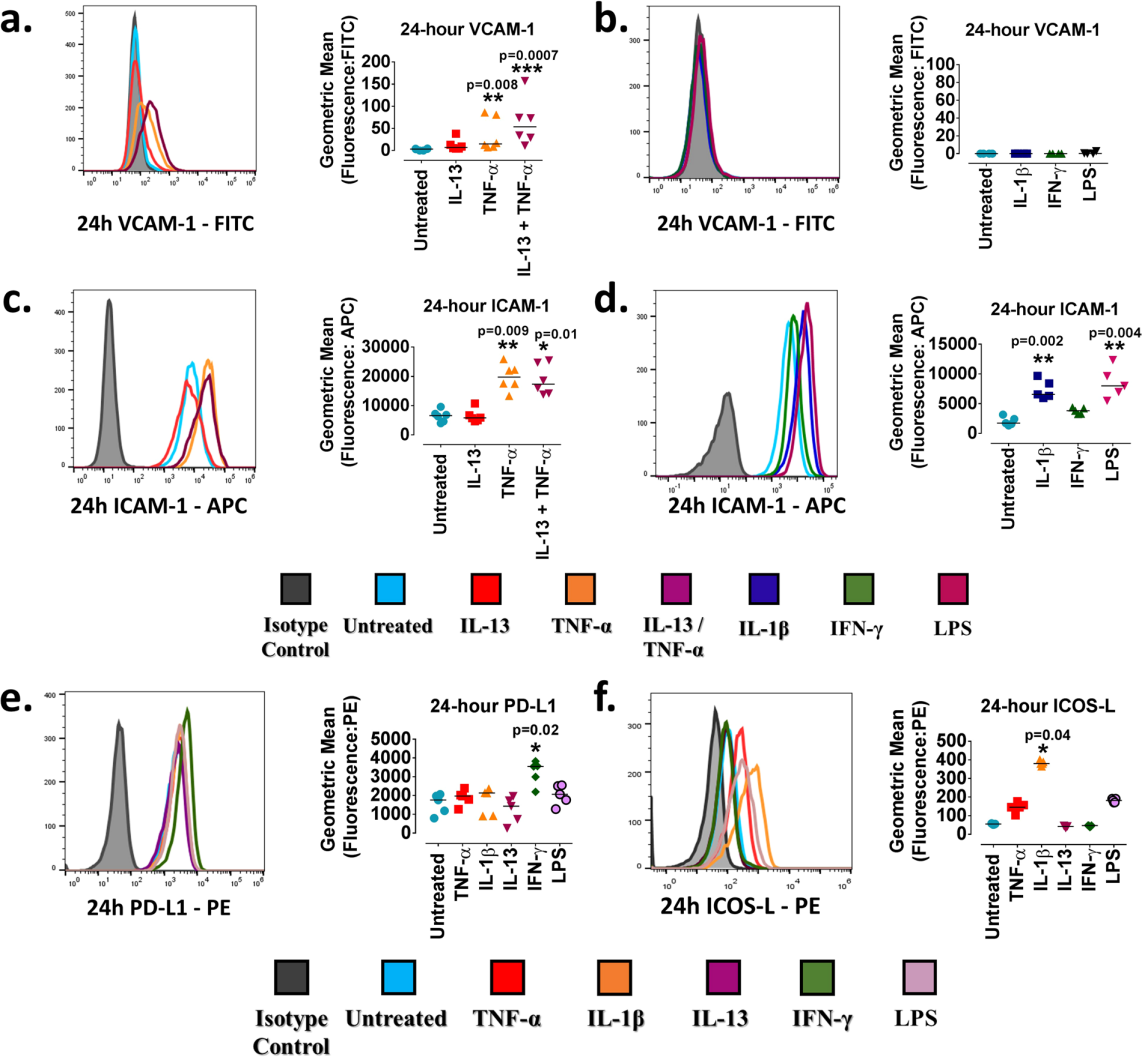
VCAM-1, ICAM-1, PD-L1 and ICOS-L surface expression following 24-hour stimulation with cytokines and LPS. **(a**) 24-hour VCAM-1 surface expression following IL-13 and TNF-α treatments. (**b**) 24-hour VCAM-1 surface expression following IL-1β, IFN-γ and LPS treatments. (**c**) 24-hour ICAM-1 surface expression following IL-13 and TNF-α treatments. (**d**) 24-hour ICAM-1 surface expression IL-1β, IFN-γ and LPS treatments. (**e**) 24-hour PD-L1 surface expression following TNF-α, IL-1β, IL-13, IFN-γ and LPS treatments. (**f**) 24-hour ICOS-L surface expression following TNF-α, IL-1β, IL-13, IFN-γ and LPS treatments. N = 4–6/group. Data presented as median [range] are analysed using Kruskal-Wallis test with Dunn’s post-hoc test. *P </= 0.05, **P < 0.01, ***P < 0.001. N = 4–6/group.

TNF-α and IL-13, alone or combined, had no significant effect on VCAM-1 and ICAM-1 surface expression at 4 h (data not shown) in contrast to 24 h, where TNF-α (15 [7–86], p = 0.008) and more robustly the combination of IL-13 and TNF-α (54 [11–157], p = 0.0007) significantly upregulated surface VCAM-1 compared to untreated ciGEnCs (3 [0–5]) (Fig. 5a). At 24 h, TNF-α significantly upregulated surface expression of the constitutively expressed ICAM-1 (19,789 [13,348–25,897], p = 0.009) (Fig. 5c) compared to untreated cells (6,609 [3,984–9,632]) but IL-13 had no effect on ICAM-1. IL-1β and LPS treatments had an effect predominantly on ICAM-1. IL-1β and LPS significantly increased surface ICAM-1 at 4 h (data not shown) compared to untreated cells, and this effect was sustained and further enhanced after 24 h (IL-1β: (6,562 [5,953–9,673], p = 0.004), LPS: (7,997 [5,497–12,334], p = 0.002) compared to untreated cells (1,727 [1,339–3,161) (Fig. 5d). No surface VCAM-1 upregulation by IL-1β, IFN-γ and LPS was observed at 24 h (untreated ciGEnC measurements were lower than those of the isotype control and therefore have been assigned 0-values) (Fig. 5b). E-/P-selectin surface expression was not modified by cytokine treatments (Supplementary Fig. 2). IFN-γ had no prominent effect on ICAM-1 or VCAM-1 but significantly increased the surface expression of PD-L1 (IFN-γ: 3,549 [2,199–3,828], p = 0.02), a negative T-cell co-stimulatory molecule^21^, after 24 h compared to untreated ciGEnCs (1,763 [793–2,067]) (Fig. 5e). Finally, surface ICOS-L expression was significantly increased only at 24 h by IL-1β (380 [365–401], p = 0.04) compared to untreated ciGEnCs (55 [52–59]) (Fig. 5f).

### TNF-α promotes neutrophil adhesion to ciGEnCs

**Figure 6:**
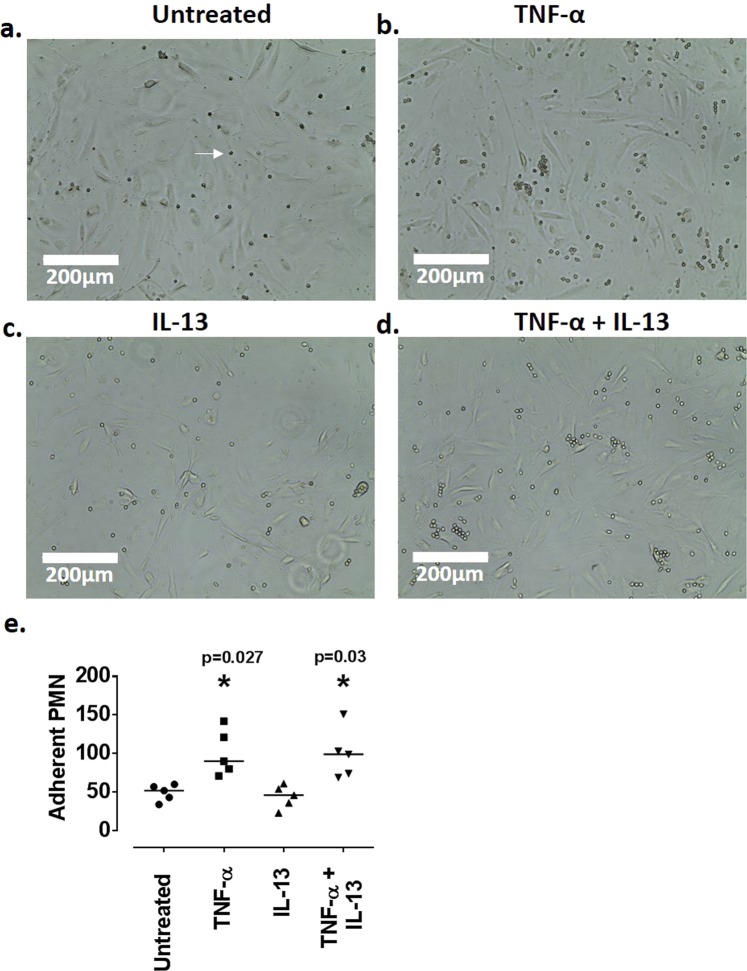
Neutrophil adhesion assay following 24 h stimulation of ciGEnCs with TNF-α and IL-13. **(a**) Neutrophil adhesion on untreated ciGEnCs. (**b**) Neutrophil adhesion on TNF-α-treated ciGEnCs. (**c**) Neutrophil adhesion on IL-13-treated ciGEnCs. (**d**) Neutrophil adhesion on TNF-α + IL-13-treated ciGEnCs. White arrow on (**a**) indicates neutrophil. (**e**) Graph of statistical analysis of neutrophil adhesion assay. Data presented as median cell number [range] are analysed using Kruskal-Wallis test with Dunn’s post-hoc test. *P </= 0.05 vs untreated. N = 6/group. Scale bars: 200 μm.

Adhesion of human neutrophils on ciGEnCs was tested following 24 h incubation of ciGEnCs with TNF-α and IL-13 alone, or in combination. Following 24 h incubation, the numbers of neutrophils bound on the ciGEnCs were counted (Fig. 6a–d). TNF-α ciGEnC treatment promoted the adhesion of a statistically significantly higher number of neutrophils ([90 cells [71–142], p = 0.027) (Fig. 6b) compared to untreated ciGEnCs (52 cells [34–60]) (Fig. 6a, 6e). The effect of the combined TNF-α and IL-13 treatments did not differ from that of TNF-α alone and led to ciGEnC adhesion of a statistically significantly higher number of neutrophils ([99 cells [69–151], p = 0.03), compared to the untreated ciGEnCs (52 cells [34–60]) (Fig. 6d, 6e). Treatments of ciGEnCs with IL-13 [46 cells [23–61]) did not increase neutrophil adhesion compared to untreated ciGEnCs (52 cells [34–60]) (Fig. 6c, 6e).

### Pro-inflammatory protein release is occurring through NF-κB and STAT1 induction in ciGEnCs

**Figure 7:**
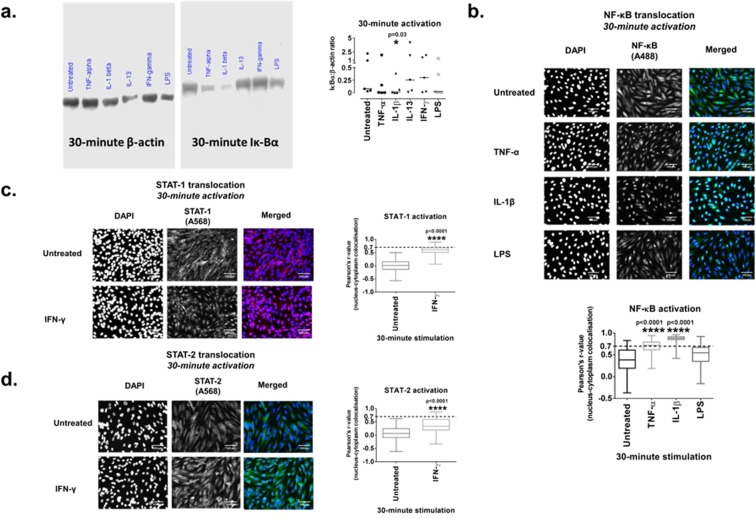
Western blotting for Iκ-Bα protein expression, 20x immunofluorescence images of ciGEnCs and diagrams of statistical analysis for NF-κB, STAT-1 and STAT-2 activation and nuclear translocation, after 30 minutes of stimulation with cytokines and LPS. (**a**) Representative image and graph of Iκ-Bα and beta-actin Western blots (blots have been cropped), following 30-minute stimulation. Data presented as (Mean +/− SEM) in ratio diagrams are analysed using Friedman test with Dunn’s post-hoc test, *P < 0.05 vs untreated. (**b**) Treatments of ciGEnCs with TNF-α, IL-1β and LPS for 30 minutes for NF-κB nuclear translocation. (**c**) Treatments of ciGEnCs with IFN-γ for 30 minutes for STAT-1 nuclear translocation. (**d**) Treatments of ciGEnCs with IFN-γ for 30 minutes for STAT-2 nuclear translocation. Data (r-values for nuclear co-localisation of DAPI and A488 or A568 for each treatment group) representative of three similar experimental repeats are presented as box and whisker plots and are analysed using Kruskal-Wallis test with Dunn’s post-hoc test. ****P < 0.0001 vs untreated. Scale bars: 100 μm.

In order to determine whether the effects observed in ciGEnCs following cytokine and LPS treatments are mediated through the NF-kB pathway, we initially used an immunoblot assay to determine nuclear factor of kappa light polypeptide gene enhancer in B-cells inhibitor, alpha (Iκ-Bα) degradation, as Iκ-Bα is the cytoplasmic inhibitor of NF-κΒ that prevents its nuclear translocation^22^. At 30 min, it was mainly IL-1β and, to a lesser extent, TNF-α, which were able to induce Iκ-Bα degradation (Fig. 7a).

Nuclear translocation of NF-kB and of signal transducer and activator of transcription (STAT)-1 and -2 (STAT-1 and STAT-2) was tested via immunofluorescence to determine pathway activation. TNF-α and IL-1β promoted NF-κB nuclear translocation at 30 min with IL-1β displaying a more robust effect than TNF-α, whereas LPS only induced low levels of NF-κB activation (Fig. 7b). IFN-γ induced STAT-1 nuclear translocation at 30 min (Fig. 7c) while it induced only moderate to low STAT-2 activation (Fig. 7d).

## Discussion

LN is one of the main and most significant complications of JSLE. LN affects the majority of JSLE patients (approx. 80%)^5–7^, and can cause severe renal damage^3^ and GFB impairment^4^. This study aimed to investigate the potential role of the human glomerular endothelium in LN inflammation using a cytokine-based *in vitro* model of LN.

The ciGEnCs were treated with cytokines previously found by our laboratory and by other studies to be elevated in plasma and/or serum samples of JSLE patients with LN (IFN-α^23^, IFN-γ^24^, TNF-α^24,25^, IL-1β^24^, IL-6^24^, IL-13^24^ and VEGF^24,26^) or shown by other studies to be expressed in the glomerular renal tissue of JSLE or adult patients with LN (IFN-γ^27^), or to have certain polymorphisms associated with JSLE (IL-1β^27,28^). The potential effect of bacterial endotoxin inflammation was also examined via 1 μg/ml LPS treatment, which, in murine models of LN and in human endotoxemia, is known to play a critical role in LN exacerbation after a bacterial infection^28–31^.

Our findings indicate that in line with *in vitro* models using HUVECs^17,32,33^ and HBMECs^34–36^, upon activation with TNF-α, IL-1β, IL-13, IFN-γ and with LPS, ciGEnCs significantly increase the production of key pro-inflammatory proteins. These included: cytokines, chemokines, blood cell growth factors, adhesion molecules and T-cell co-stimulatory molecules. All proteins demonstrated in this study to be upregulated in human ciGEnCs have been previously shown to be involved in human LN or other types of human renal disease and are summarised on Table 1.

**Table 1:** Proteins produced by activated ciGEnCs and their involvement in renal disease.

Proteins induced by TNF-α, IL-1β, IL-13, IFN-γ and LPS	Involvement in SLE and renal disease
MCP-1	MCP-1 expression within the glomerulus can be predictive of poor outcomes in JSLE patients with LN^37^.
VCAM-1	VCAM-1 serum and urinary levels have been shown to be upregulated and to be associated with active LN^69,70^.
ICAM-1	ICAM-1 urinary levels are upregulated in patients with class III, IV and V LN^71^.
IL-6	High IL-6 levels have been detected in the urine of active LN patients^72^.
IL-8	High IL-8 levels have been detected in the urine of active LN patients^72^.
IL-10	Plasma IL-10 has been found to be significantly elevated in JSLE patients with active disease^73^.
M-CSF	Serum and urine M-CSF levels have been shown to be a reliable and sensitive biomarkers for LN^74,75^.
GM-CSF	Increased mRNA levels of GM-CSF are expressed by *in vitro* cultured human renal tubular cells derived from interstitial fibrotic kidneys compared to tubular cells derived from non-fibrotic kidneys^76^.
MIP-1α	Together with MCP-1, MIP-1α promotes macrophage recruitment and activation in the kidneys of patients with crescentic GN^77^.
IP-10	IP-10 serum levels are upregulated in LN patients^78^.
TNF-α	TNF-α gene polymorphisms leading to excessive serum TNF-α production can predispose patients to LN development^79^.
IFN-γ	Significantly increased IFN-γ positive immune-histochemical staining has been observed in biopsies from juvenile-onset LN patients compared to HCs^27^.
PD-L1	In JSLE, decreased PD-L1 expression by antigen-presenting cells has been associated with active disease^80^.
ICOS-L	ICOS-L plasma levels are increased in patients with active SLE compared to those with inactive SLE^81^.

Single TNF-α and IL-1β treatments upregulated MIP-1α and GM-CSF and combined TNF-α and IL-1β treatment significantly increased MCP-1 secretion. LPS also had a prominent effect in MIP-1α and GM-CSF secretion. MCP-1 promotes infiltration by inflammatory dendritic cells in LN^37^ and MIP-1α can activate and attract human granulocytes, macrophages and monocytes^38^ whereas GM-CSF promotes the maturation and differentiation of macrophages, neutrophils, eosinophils and basophils^39^. IL-1β and LPS also upregulated IL-6 and TNF-α secretion. IL-6 promotes B- and T-cell differentiation^40^ whereas TNF-α is a prototypical pro-inflammatory cytokine^41^ affecting a broad variety of cell types, including the human endothelium.

IFN-γ increased the secretion of IP-10, IL-10 and TNF-α, as did LPS. IP-10 mediates T-cell accumulation to the inflamed tissues^42^ whereas IL-10 is involved in autoantibody production^43^. The combined cytokine treatment, which could be mainly attributed to TNF-α, IL-1β and IFN-γ led to increased amounts of secreted M-CSF as the other cytokines had minimal effects individually. When the combined effect of the prototypical pro-inflammatory cytokines -TNF-α and IL-1β- was specifically tested, the ciGEnCs were found to significantly upregulate M-CSF secretion compared to the untreated ciGEnCs. M-CSF promotes the proliferation, differentiation, and survival of monocytes and macrophages^44^. LPS had a prominent effect on secretion of IFN-γ which is involved in anti-viral immunity^45^. TNF-α and IL-1β appeared to increase secretion of IL-8, a potent neutrophil chemokine^46^ although not to a level of statistical significance tested. These data suggest that GEnCs secrete mediators into the environment that induce the recruitment, activation and maturation of inflammatory leukocytes leading to perpetuation of the inflammatory response.

Two important adhesion molecules were found to be upregulated by the ciGEnCs following pro-inflammatory stimulation; VCAM-1 and ICAM-1. VCAM-1 is involved in the binding of lymphocytes, monocytes, eosinophils, and basophils but not neutrophils to the vascular endothelium^47^ whereas ICAM-1 promotes all leukocytes’ binding to the endothelium^48^. In this study, IL-13 had an effect exclusively on soluble and surface VCAM-1 expression with the latter being promoted by IL-13 in combination with TNF-α. ICAM-1 expression was significantly increased by TNF-α, IL-1β and LPS. 24 h ICAM-1 upregulation by TNF-α, IL-1β and LPS has also been confirmed in HUVECs^49,50^. Similar to the human ciGEnCs and in contrast to other types of human endothelial cells (carotid, coronary), lack of 24 h IL-1β- and LPS-mediated upregulation of surface VCAM-1 has been previously observed in HUVECs and human subclavian endothelial cells^50^.

The differential cytokine regulation of surface VCAM-1 and ICAM-1 expression could imply that certain pro-inflammatory stimuli could lead to preferential intra-renal accumulation of certain immune cell populations. Indeed, our findings from the neutrophil adhesion assay indicated that combinatorial TNF-α and IL-13 treatment of ciGEnCs had an effect similar to that of TNF-α alone and led to ciGEnC adhesion of similar numbers of neutrophils, which were significantly higher than those adhering to untreated or IL-13-treated ciGEnCs. Thus, ICAM-1 that was found in this study to be induced by TNF-α but not IL-13 (IL-13 had an effect only on VCAM-1 expression), could be the adhesion molecule predominantly responsible for neutrophil binding on ciGEnCs. This assumption is further corroborated by evidence in the literature that, in contrast to VCAM-1, ICAM-1 can promote binding of neutrophils on the endothelium^48^.

In this study an attempt was made to assess the expression of E/P-selectin by the ciGEnCs in response to our pro-inflammatory model however no expression was seen. A recent study demonstrated that GEnCs do not require E/P-selectin for neutrophil binding due to an increased dwell time by cells at this location^51^. This has been confirmed both in mouse models using lupus-prone mice and in human renal biopsies from 108 patients with primary renal disease and allograft recipients^52–55^.

PD-L1 surface expression that was upregulated at 24 h in ciGEnCs by IFN-γ treatment, can prevent the activation of CD-8^+^ T-cells^21^ and inhibit the autoreactive T-cell function^56^. However, after 24 h, IL-1β significantly increased surface expression of the positive CD4^+^ T-cell co-stimulatory molecule, ICOS-L^57^. Therefore, T-cell-driven kidney inflammation could be promoted by CD4^+^ instead of CD8^+^ T-cells.

Incubation of ciGEnCs for 4 h and 24 h reflects acute phase inflammation^58^ rather than chronic inflammation. The 24 h cytokine and LPS stimulation did not significantly induce apoptotic or necrotic cell death, indicating that during acute inflammation human ciGEnCs maintain their integrity and acquire a pro-inflammatory phenotype due to cell activation and not due to cell death. Downstream activation of the central pro-inflammatory transcription factor NF-κB primarily occurred following IL-1β and TNF-α stimulation and to a lesser extent by LPS and was demonstrated by nuclear NF-κB translocation as well as by Iκ-Bα degradation. In contrast, IFN-γ was found to activate downstream STAT-1 signalling but not STAT-2 which is normally activated by IFN-α.

NF-κB shRNA knock-down in human microvascular endothelial cells (HMECs) has been shown to lead to strong suppression of the expression of TNF-α-induced genes such as *MCP-1*, *M-CSF*, *GM-CSF*, *IL-6* and *IL-8*^59^. Furthermore, STAT-1 siRNA knock-down in IFN-γ-treated HUVECs has been shown to downregulate secretion of IP-10 and other T-cell related chemokines^60^ providing supporting evidence that these pathways are activated in endothelial cells. Thus, it could be hypothesised that in human GEnCs similar mechanisms of action could be occurring.

The 24 h serum treatments did not demonstrate significant changes in the mRNA expression levels of most pro-inflammatory genes tested. However, *IL-6* and *IL-1β* mRNA levels were increased by the JSLE sera. In addition, IL-6 secretion by the ciGEnCs underwent a pronounced increase after both active and inactive LN serum treatments. On the contrary, IL-1β secretion by ciGEnCs was not affected by either of the serum treatments. This finding suggests potential lack of inflammasome and caspase-1 activation in ciGEnCs following serum treatments which could lead to a reduction in the release of active IL-1β within the microenvironment.

The limited amount of blood samples due to rarity of the disease as well as the small blood sample volumes obtained from paediatric patients did not allow for further investigation and detection of the JSLE serum factors inducing the expression IL-6 and IL-1β production. A potential candidate, however, could be autoantibodies present in the sera samples. Indeed, previous research studies using affinity purified IgG antibodies from anti-endothelial cell antibody (AECA)-positive SLE patients to treat HUVECs *in vitro*, demonstrated increased HUVEC-derived secretion of IL-1^61^ and IL-6^62^. Similar findings have also been observed in scleroderma and systemic vasculitis patients^62,63^.

The lack of strong responses induced by the JSLE sera may be due to the immunosuppressant regimens followed by patients. Increasing the serum doses used in these experiments may be required to demonstrate an effect; however, the 5% treatments (limited due to sample volumes from paediatric patients) did not suffice to induce a strong *in vitro* ciGEnC pro-inflammatory response.

Furthermore, the serum treatments could have had a more pronounced effect on ciGEnCs in an *in vitro* model in which ciGEnCs would be co-cultured with human podocytes and/or mesangial cells. Lack of crosstalk between native glomerular renal cells could be affecting the results obtained from the serum treatments. The development of a co-culture model, however, was beyond the scope of this study, the aim of which was to investigate pro-inflammatory properties of the human ciGEnCs, a type of human endothelial cells that has been little studied.

There are inherent limitations with using a cell line to generate a model of human disease. However, ciGEnCs retain all the morphological and functional characteristics of the primary GEnCs and express at levels comparable to those of primary GEnCs, the majority of endothelial cell-specific markers^64^. These characteristics render the ciGEnCs into a reliable tool for the study of human GEnCs. Future work could focus on validating these findings using primary cells or in an *in vivo* murine model however this was beyond the scope of this study.

**Figure 8:**
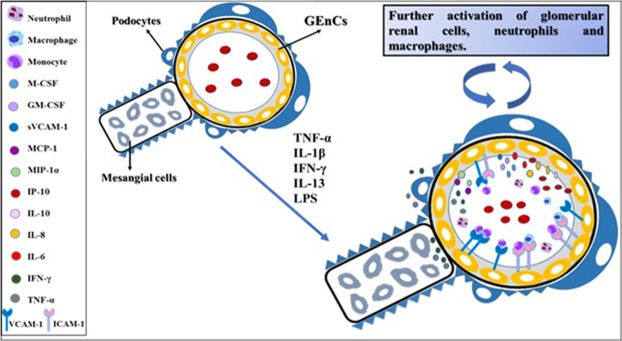
Potential effect of stimulated GEnCs in LN based on the *in vitro* findings. Under the highly inflammatory environment created within the glomeruli by the presence of TNF-α, IL-1β, IL-13 and IFN-γ but also by the potential presence of LPS, the human GEnCs will be activated to produce, secrete and express a variety of pro-inflammatory cytokines, chemokines, blood cell growth factors and adhesion molecules that will further exacerbate the renal disease by either promoting the infiltration of immune cells within the glomeruli or by activating their neighbouring renal cells, the podocytes and mesangial cells.

In conclusion, our data imply that the glomerular endothelium could have a pivotal role in LN inflammation (Fig. 8). As demonstrated by our *in vitro* model, the GEnCs are not just passive bystanders but actively respond to the renal pro-inflammatory environment created in kidney disease.

## Materials and Methods

### Materials

Recombinant cytokines were purchased from Peprotech, LPS was purchased from Sigma. All antibodies are listed on Supplementary Table 1.

### Methods

All methods reported here were carried out in accordance with relevant guidelines and regulations of the University of Liverpool. All experimental protocols were reviewed and approved by either the North West – Liverpool East Research Ethics Committee (UK JSLE Cohort Study and Repository) (REC: 06/Q1502/77) or the University of Liverpool Committee of Research Ethics (Adult Healthy Control samples) (Research Ethics Approval Number: RETH000773). Informed consent was obtained from all subjects or, if subjects are under 18, from a parent and/or legal guardian.

### Human conditionally immortalised glomerular cell line culture

The ciGEnCs were developed and kindly donated by Professor Moin Saleem and Dr Simon Satchell (Children’s Renal Unit, Bristol Royal Hospital for Children)^64^. The ciGEnCs were cultured at 33 °C (with 5% CO_2_) in endothelial growth medium 2-microvascular (EGM-2 MV Bulletkit, CC-3202, Lonza) containing 5% Fetal Bovine Serum (FBS) and growth factors as supplied (VEGF was excluded as it has been utilised for treatments). Once cells reached 50–70% confluence they were thermoswitched at 37 °C (with 5% CO_2_) for seven days until complete differentiation was achieved and confirmed via light microscopy.

For all experiments, a minimum of three consecutive ciGEnC passages was used for reproducibility purposes. Consecutive passages between 29 and 35 were used, as the researchers who developed and donated the ciGEnCs had previously shown that ciGEnCs can retain their primary GEnC-specific morphological and functional characteristics up to passage 41^64^. All changes in mRNA expression or protein expression, secretion and production levels were compared to those of the untreated ciGEnCs. The fully differentiated ciGEnCs were incubated at 37 °C for 30 mins, 4 h and 24 h with 10 ng/ml (a concentration previously used to induce pro-inflammatory changes in endothelial cells *in vitro*^65–67^)- of seven human recombinant cytokines (IFN-α, IFN-γ, TNF-α, IL-1β, IL-6, IL-13, VEGF), individually and in combination (All) (cytokine concentration response assays were also performed and are presented on Supplementary Fig. 4) and with 1 μg/ml of LPS.

Fully differentiated ciGEnCs were treated for 4 h or 24 h with 5% (5% sera concentration matching the 5% FBS concentration in standard media used for ciGEnC culture) serum samples in medium without FBS, from JSLE patients with active LN (renal BILAG: A/B, N = 10) and inactive LN (renal BILAG: D/E, N = 10) and with 5% sera from age-matched healthy controls (N = 10). Following completion of treatments, qRT-PCR was performed as above to assess changes in the mRNA expression levels between ciGEnC groups treated with HC and LN sera. Patient and HC information are presented on Table 2. To test the effect of serum samples on IL-6 and IL-1β secretion by the ciGEnCs, an additional of N = 6 of active LN, inactive LN and HC serum samples were collected and used to treat the ciGEnCs for 24 h, at a 5% concentration (patient and HC information presented on Table 3).

**Table 2:** Serum treatments for changes in ciGEnC pro-inflammatory gene expression. Age, gender, ethnicity, renal BILAG scores and medications for JSLE patients; age, gender and ethnicity for paediatric healthy controls (N = 10/group).

Demographics	Active LN (10)	Inactive LN (10)	Healthy Controls (10)
Age (years) (median [range])	15 [9–17]	14.5 [11–18]	13 [9–17.2]
Age at diagnosis (years) (median [range])	12.4 [6.28–14.48]	11.73 [9.3–15.59]	—
Females (%)	90 (9)	90 (9)	70 (7)
Nationality (%)	0	0	90 (9)
White British	10 (1)	0	0
Black British	10 (1)	10 (1)	0
British	0	30 (3)	0
Indian	0	10 (1)	0
Nepalese	10 (1)	30 (3)	0
Pakistani	10 (1)	0	0
Bangladeshi	10 (1)	0	0
Chinese	40 (4)	10 (1)	0
African	0	10 (1)	0
Caucasian	10 (1)	0	10 (1)
Not stated			
Renal BILAG score (%)	30% (3)	80% (8)	—
A	70% (5)	20% (2)	
B			
D			
E			
Renal disease manifestations (%)	70 (7)	—	—
Proteinuria (ACR > 100 mg/mmol)	10 (1)	—	
Nephrotic Syndrome	10 (1)	—	
Renal Hypertension	10 (1)	—	
Low GFR (<80 ml/min/1.73 m^2^)	10 (1)	—	
Increased plasma creatinine (>130 μmol/ml)	10 (1)	—	
Urine ACR (mg/dL) (median [range])	—	80 (8)	
Previous renal involvement			
Biopsy-proven LN (%)	70 (7)	30 (3)	—
LN class (WHO or ISN/RPS) (%)	10 (1)	—	
II	10 (1)	—	
III/IV	—	30 (3)	
IV	10 (1)	—	
IV-C	20 (2)	—	
V	10 (1)	—	
IV/V	10 (1)	—	
Mixed			
Medications dosage [range] (patient n-number)	200 mg [200–700] (7)	200 mg [200–300] (7)	—
Hydroxychloroquine	150 mg [100–300] (3)	100 mg [100–100] (2)	
Azathioprine	1500 mg [1200–2000] (5)	1500 mg [750–2000] (6)	
Mycophenolate mofetil	7.5 mg [2.5–45] (6)	10 mg [2–10] (6)	
Prednisolone	—	20 mg [17.5–22.5] (2)	
Methotrexate (oral)	2000 mg (1), 6 pulses (1)	—	
Rituximab	520 mg/m^2^ (1)	—	
Cyclophosphamide	—	2 g (1)	
IVIG

**Table 3:** Serum treatments for changes in ciGEnC IL-1β and IL-6 secretion. Age, gender, ethnicity, renal BILAG scores and medications for JSLE patients; age, gender and ethnicity for paediatric healthy controls (N = 6/group).

Demographics	Active LN (6)	Inactive LN (6)	Healthy Controls (6)
Age (years) (median [range])	15.53 [12.18–16.58]	14.55 [11.03–17.79]	14.9 [12.12–16.6]
Age at diagnosis (years) (median [range])	12.8 [6.28–13.29]	10.46 [6.28–16.88]	—
Females (%)	100 (6)	100 (6)	100 (6)
Nationality (%)	16.6 (1)	33.3 (2)	100 (6)
White British	16.6 (1)	16.6 (1)	0
Chinese	33.3 (2)	33.3 (2)	0
Somali	16.6 (1)	16.6 (1)	0
African	16.6 (1)	0	0
Indian			
Renal BILAG score (%)	16.7% (1)	66.7% (4)	—
A	83.3% (5)	33.3% (2)	
B			
D			
E			
Renal disease manifestations (%)	16.6 (1)	0	—
Renal Hypertension (%)	239.3 [0.7–592.4]	7.5 [0.8–8.8]	
Urine ACR (mg/dL) (median [range])	45 [37–62]	51 [30–61]	
Renal Creatinine (mg/dL) (median [range])	138 [99.8–158.1]	121.5 [99.1–181.9]	
Estimated GFR (mL/min/1.73 m²) (median [range])			
Medications (patient n-number)	4	3	—
Hydroxychloroquine	0	3	
Azathioprine	6	3	
Mycophenolate mofetil	5	5	
Prednisolone	0	0	
Methotrexate (oral)	1	0	
Rituximab	1	0	
Cyclophosphamide			

### qRT-PCR

RNA was extracted using Trizol-chloroform and in-column DNase digestion (Qiagen RNeasy Mini Kit). RNA concentrations and purity were determined using Nanodrop (ND-1000 Spectrophotometer, Thermo Fisher Scientific). 200 ng RNA was transcribed into cDNA using the AffinityScript multi-temp cDNA synthesis kit (Agilent Technologies, Cheshire, UK) as per manufacturer’s instructions. qRT-PCR was performed using primers described in Supplementary Table 2 with the Brilliant III Ultra-fast SYBR QPCR mastermix kit (Agilent Technologies) as per manufacturer’s instructions. mRNA expression for target genes was normalised using the mean (for one housekeeping (HK) gene) or the geometric mean (for more than one HK genes) of the following HK genes: beta-actin (ACTB), TATA-binding protein (TBP), beta-tubulin (TUBB). The ΔΔCt value was calculated as follows:

$${\rm{\Delta }}{\rm{\Delta }}\mathrm{Ct}={2}^{-({\bf{Mean}}{\bf{Ct}}[{\rm{target}}{\rm{gene}}]-({\bf{Geo}}){\bf{Mean}}{\bf{Ct}}[{\rm{HK}}\mathrm{gene}(s)]}.$$

### ELISA assays

The secretion of MCP-1, sVCAM-1, IL-6, IL-8, IL-10, M-CSF, GM-CSF, MIP-1α, IP-10, TNF-α and IFN-γ was tested with single ELISAs (R&D Systems DuoSets) or a Luminex multiplex ELISA assay, utilising conditioned media from ciGEnCs treated for 24 hours with cytokines (as above) and LPS. Conditioned media from IL-6 and VEGF treatments were excluded as they induced no change in the mRNA expression of the urinary biomarkers. However, IL-6 and VEGF were still included in the combined cytokine treatment (All).

### Flow cytometry

For VCAM-1, ICAM-1, E-/P-selectin, PD-L1 and ΙCOS-L detection, the ciGEnCs were stimulated for 24 and then were stained for 30 min on ice in the dark with fluorescence-conjugated antibodies against VCAM-1-FITC, ICAM-1-APC, E-/P-selectin-PE, PD-L1-PE and ICOS-L-PE with appropriate isotype controls. Apoptosis was assessed using the Annexin V/PI kit (Sigma) as per manufactureur’s instructions. Flow cytometry was performed using a Merck Guava Flow Cytometer and data were analysed using FlowJo 10.3 software.

### Western blotting (WB)

For the WB experiments, ciGEnCs were stimulated for 30 min, proteins were extracted using RIPA buffer and protein concentrations were determined using the Pierce™ BCA Protein Assay Kit (Thermo Fisher Scientific). 15 μg of protein was diluted in NuPAGE LDS Sample buffer (4x, Invitrogen) at a final 1x concentration with 50 μM dithiothreitol (DTT, Sigma-Aldrich). The protein samples were loaded into 50 μl 12% 10-well precast polyacrylamide gels and transferred to PVDF membranes (Bio-Rad) using the Trans-Blot Turbo Transfer System (Bio-Rad). Antibodies were used for the detection of human beta-actin (β-actin) and Iκ-Bα. Following gel transfer, membranes were blocked in TBS-T with 5% milk for 1 h, were incubated with the primary antibodies overnight at 4 °C, washed in TBS-T and incubated with secondary antibodies for 1 h (room temperature). Protein detection was achieved using enhanced chemiluminescence (ECL) reagents (Li-COR). Li-COR software was used for protein band densitometry. Due to β-actin and Iκ-Bα having a similar molecular weight, the samples were loaded and were run twice onto two different gels, which were blocked for either β-actin or Iκ-Bα (full-length images are presented on Supplementary Fig. 3).

### Neutrophil adhesion assay

ciGEnCs were stimulated with 10 ng/ml of TNF-α and IL-13, alone or in combination, for 24 h (untreated ciGEnCs were included). Whole-blood from adult healthy controls was used to isolate human neutrophils via HetaSep (StemCell)-aided separation of white blood cells from red blood cells and subsequent Histopaque (Sigma-Aldrich) centrifugation of white blood cells. Neutrophils were re-suspended in RPMI (+10% FBS) at a concentration of 3 × 10^6^/ml. ciGEnCs were then washed in PBS and following PBS removal 1 ml of neutrophil suspension was added onto each well. Cells were then incubated for 90 min at 37 °C. Following 90 min incubation, conditioned media containing non-adherent neutrophils were collected and neutrophils were counted using a cell counter. The cells remaining on the plate were washed in PBS and images were taken at 20x magnification. 5 random images per well were collected and the number of neutrophils adherent to the ciGEnC monolayer was counted using ImageJ software.

### Immunofluorescence (IF)

Transcription factor activation and nuclear translocation was assessed using IF. Following 30 min incubation, cells were fixed in 4% formaldehyde for 30 mins and permeabilised using 0.4% Triton-X-100 for 10 mins. Non-specific binding was blocked using PBS-1% BSA for 1 h (room temperature). Antibodies against human NF-κΒ, STAT-1 and STAT-2 were added overnight, at 4 °C. ciGEnCs were washed three times with PBS and incubated with fluorescence-conjugated secondary antibodies and 1 μg/ml of DAPI (4′,6-diamidino-2-phenylindole), for 2 h at room temperature. Cells were visualised immediately, using the EVOS FLoid Cell Imaging Station (ThermoFisher Scientific).

### Image analysis

Image analysis to determine nuclear translocation of the signal in response to cytokine or LPS treatment was performed using Fiji (ImageJ) and particularly the Coloc2 algorithm^68^. For every group of treatments, the Pearson’s r-values for every single cell, representing the degree of nuclear colocalisation between DAPI and A488 or A568, were obtained.

### Statistical analysis

Statistical analysis of all data was performed using GraphPad Prism 4.0 software. Data are expressed as median values[range]. Multiple comparisons were made using Kruskal-Wallis or Friedman non-parametric test with Dunn’s post-hoc test for three or more treatment groups and Mann-Whitney non-parametric test for two treatment groups. Statistical significance was set a priori at a p value < 0.05. For the IF assay, the r-values from each treatment group were used to perform non-parametric Kruskal-Wallis statistical test (with Dunn’s post-hoc test) for three or more treatment groups, or Mann-Whitney tests for two treatment groups. For the nuclear colocalisation to be statistically significant, the r-values should range from 0.7 to 1 (0.7 indicated by dashed line in graphs).

## Supplementary information

Supplementary Figures and Tables
