# Supplementary material for: The human glomerular endothelial cells are potent pro-inflammatory contributors in an in vitro model of lupus nephritis
Source: Sci Rep. 2019 Jun 6;9:8348. doi: 10.1038/s41598-019-44868-y (PMC6554346; doi:10.1038/s41598-019-44868-y)
Supplement: Supplementary file 1 — Supplementary Figures and Tables [file 41598_2019_44868_MOESM1_ESM.docx]

**The human glomerular endothelial cells are potent pro-inflammatory contributors in an *in vitro* model of lupus nephritis**

**Paraskevi Dimou, PhD^1^, Rachael D Wright, PhD^1^, Kelly L Budge, BA^1^, Angela Midgley, PhD^1^, Simon C Satchell, PhD^3^, Matthew Peak, PhD^2^ and Michael W Beresford, PhD^1,2,4^**

^1^Department of Women’s and Children’s Health, Institute of Translational Medicine, University of Liverpool, UK

^2^NIHR Alder Hey Clinical Research Facility, Alder Hey Children’s NHS Foundation Trust, Liverpool, UK

^3^Academic Renal Unit, University of Bristol, Bristol, UK

^4^Department of Paediatric Rheumatology, Alder Hey Children’s NHS Foundation Trust, Liverpool. UK

To whom correspondence should be addressed:

Professor Michael W Beresford,

Institute in the Park, Alder Hey Children’s NHS Foundation Trust,

Eaton Road, Liverpool, L12 2AP, UK.

Tel.: +44 151 282 4536; Email: m.w.beresford@liverpool.ac.uk

Running Headline: **Dimou *et al***

**Supplementary Figure and Table Legends**

Supplementary Table 1. Synopsis of proteins produced by cytokine- and LPS-mediated stimulation of ciGEnCs.

**Supplementary Table 2.** List of antibodies.

**Supplementary Table 3.** List of primers.

Supplementary Figure 1. ciGEnC viability after 24 hours and 4 days of continuous TNF-α, IL-1β, IL-13, IFN-γ and LPS exposure. a. Viable (Anx V-/PI-), early apoptotic (Anx V+/PI-) and late apoptotic/necrotic (Anx V+/PI+) ciGEnCs after 24 hours of exposure to cytokines and LPS. N=5/group. b. Representative flow cytometric diagram.

**Supplementary Figure 2. Full-length images of western blot gels for β-actin and Iκ-Bα expression after 30 minutes of cytokine and LPS stimulation. a.** β-actin. **b.** Iκ-Bα. Unstimulated PMBCs were used as WB antibody controls.

Supplementary Figure 3. E-/P-selectin surface expression following 4- and 24-hour stimulation with TNF-α and IL-13. a. 4-hour E-/P-selectin surface expression following IL-13 and TNF-α treatments. b. 24-hour E-/P-selectin surface expression following IL-13 and TNF-α treatments. Data presented as median [range] are analysed using Kruskal-Wallis test with Dunn’s post-hoc test, p=NS.

**Supplementary Figure 4. Concentration response for a.** TNF-α, **b.** IL-1β, **c.** IL-13 and **d.** IFN-γ.

Supplementary Table 1

| **FC Antibody (anti-human)** | **Clone** | **Working Concentration** | **Catalogue Number, Company** |
| --- | --- | --- | --- |
| VCAM-1 - FITC (Mouse IgG1, kappa) | STA | 4 μg/ml | 11-1069, eBioscience/Affymetrix |
| ICAM-1 - APC (Mouse IgG1, kappa) | HA58 | 1 μg/ml | 17-0549, eBioscience/Affymetrix |
| PD-L1 - PE (Mouse IgG1, kappa) | MIH1 | 2 μg/ml | 12-5983-42, Invitrogen/Thermo Fisher Scientific |
| E-/P-selectin – PE (Mouse IgG1, kappa) | BBIG-E6 (13D5) | 4 μg/ml | FAB6169P, R&D Systems/Bio-Techne |
| **Isotype Control Antibody** | **Clone** | **Catalogue Number, Company** |  |
| Mouse G1 kappa- FITC | P3.6.2.8.1 | 17-4714, eBioscience/Affymetrix |  |
| Mouse IgG1 kappa - APC | P3.6.2.8.1 | 17-4714, eBioscience/Affymetrix |  |
| Mouse IgG1 kappa - PE | P3.6.2.8.1 | 14-4714-82, Invitrogen/Thermo Fisher Scientific |  |
|  |  |  |  |
| **IF Antibody (anti-human)** | **Clone** | **Working Concentration** | **Catalogue Number, Company** |
| Rabbit RelA/NF-κB p65 | Polyclonal | 400 ng/ml | NB100-2176, Novus Biologicals/Bio-Techne |
| Mouse STAT-1 | 655210 | 780 ng/ml | MAB14901, R&D Systems/Bio-Techne |
| Rabbit STAT-2 | Polyclonal | 1.25 μg/ml | 44-362G, Thermo Fisher Scientific |
| **Secondary antibody** | **Clone** | **Working Concentration** | **Catalogue Number, Company** |
| Goat anti-mouse - A568 | Polyclonal | 4 μg/ml | A-11004, Thermo Fisher Scientific |
| Goat anti-rabbit - A488 | Polyclonal | 4 μg/ml | A-11008, Thermo Fisher Scientific |
|  |  |  |  |
| **WB Antibody (anti-human)** | **Host species** | **Working Concentration** | **Catalogue Number, Company** |
| Rabbit IκBα (monoclonal) | rabbit | 100 ng/ml | 4812, Cell Signaling Technology |
| Mouse β-actin | mouse | 333.3 ng/ml | ab8226, Abcam |
| **Secondary Antibody** | **Host species** | **Working Concentration** | **Catalogue Number, Company** |
| Goat anti-mouse IgG-HRP | Goat | 20 ng/ml | HAF007, R&D Systems |
| Goat anti-rabbit IgG-HRP | Goat | 20 ng/ml | HAF008, R&D Systems |

Supplementary Table 2

| **Gene** | **Forward Primer Sequence (5'- 3')** | **Reverse Primer Sequence (5'- 3')** |
| --- | --- | --- |
|  |  |  |
| **ACTB** | CATTGCGGTGGACGATGGA | AGATCAAGATCATTGCTCCTCCTG |
| **TBP** | GTGACCCAGCATCACTGTTTC | GAGCATCTCCAGCACACTCT |
| **TUBB** | GGACCGCATCTCTGTGTACT | CTGCCCCAGACTGACCAAATA |
| **IL-6** | AGAGGCACTGGCAGAAAACA | TCACCAGGCAAGTCTCCTCA |
| **IL-8** | CAGAGACAGCAGAGCACACA | GGCAAAACTGCACCTTCACA |
| **IL-10** | GCGCTGTCATCGATTTCTTCC | GCCACCCTGATGTCTCAGTT |
| **M-CSF** | CCCCAGTCCTCTCTTAAAAGGC | GAGAGGACCCAGGCAAACTT |
| **GM-CSF** | GAGACACTGCTGCTGAGATGAA | AGGAAGTTTCCGGGGTTGGAG |
| **MCP-1** | CTCGCTCAGCCAGATGCAAT | TCTCCTTGGCCACAATGGTC |
| **VCAM-1** | GGTGGGACACAAATAAGGGT | GCTTGAGAAGCTGCAAAC |
| **MIP-1α** | CATTCCGTCACCTGCTCAGA | AGCAGCAAGTGATGCAGAGA |
| **IP-10** | AGCCCCACGTTTTCTGAGAC | GAGAGAGGTACTCCTTGAATGCC |
| **TNF-α** | CTTCTGCCTGCTGCACTTTG | GGGTTTGCTACAACATGGGC |
| **IL-1β** | CAACAGGCTGCTCTGGGATT | CCTGGAAGGAGCACTTCATCT |
| **NF-κB** | ACCCGGCTTCAGAATGGCA | AGGTATGGGCCATCTGCTGTT |

Supplementary Table 3

| **Pro-inflammatory stimulus** | **Proteins produced by activated ciGEnCs** |
| --- | --- |
| - **TNF-α** | M-CSF, GM-CSF, MIP-1α, MCP-1, Surface VCAM-1, ICAM-1 |
| - **IL-1β** | IL-6, M-CSF, GM-CSF, MIP-1α, TNF-α, MCP-1, ICAM-1, ICOS-L |
| - **IL-13** | Soluble and cell surface VCAM-1 |
| - **IFN-γ** | IL-10, IP-10, TNF-α, PD-L1 |
| - **LPS** | IL-6, IL-10, IP-10, MIP-1α, IFN-γ, MCP-1, ICAM-1 |

Supplementary Figure 1


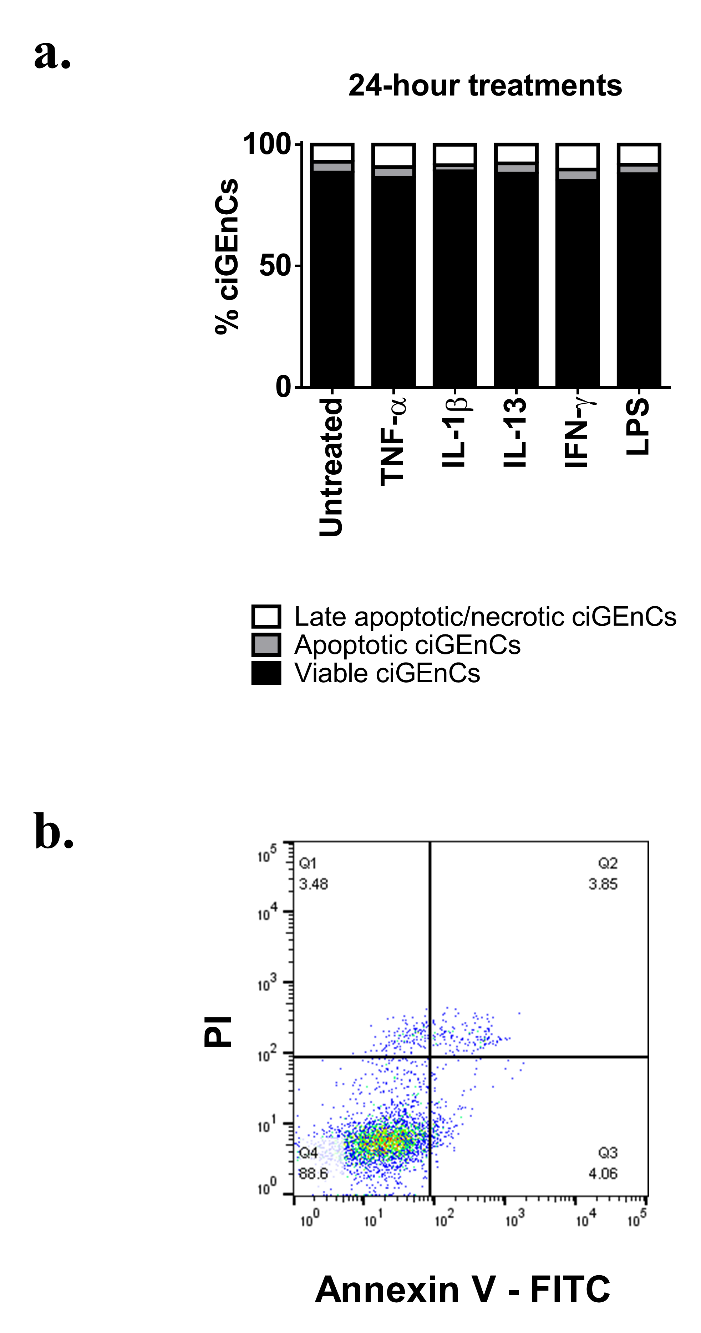


Supplementary Figure 2

**
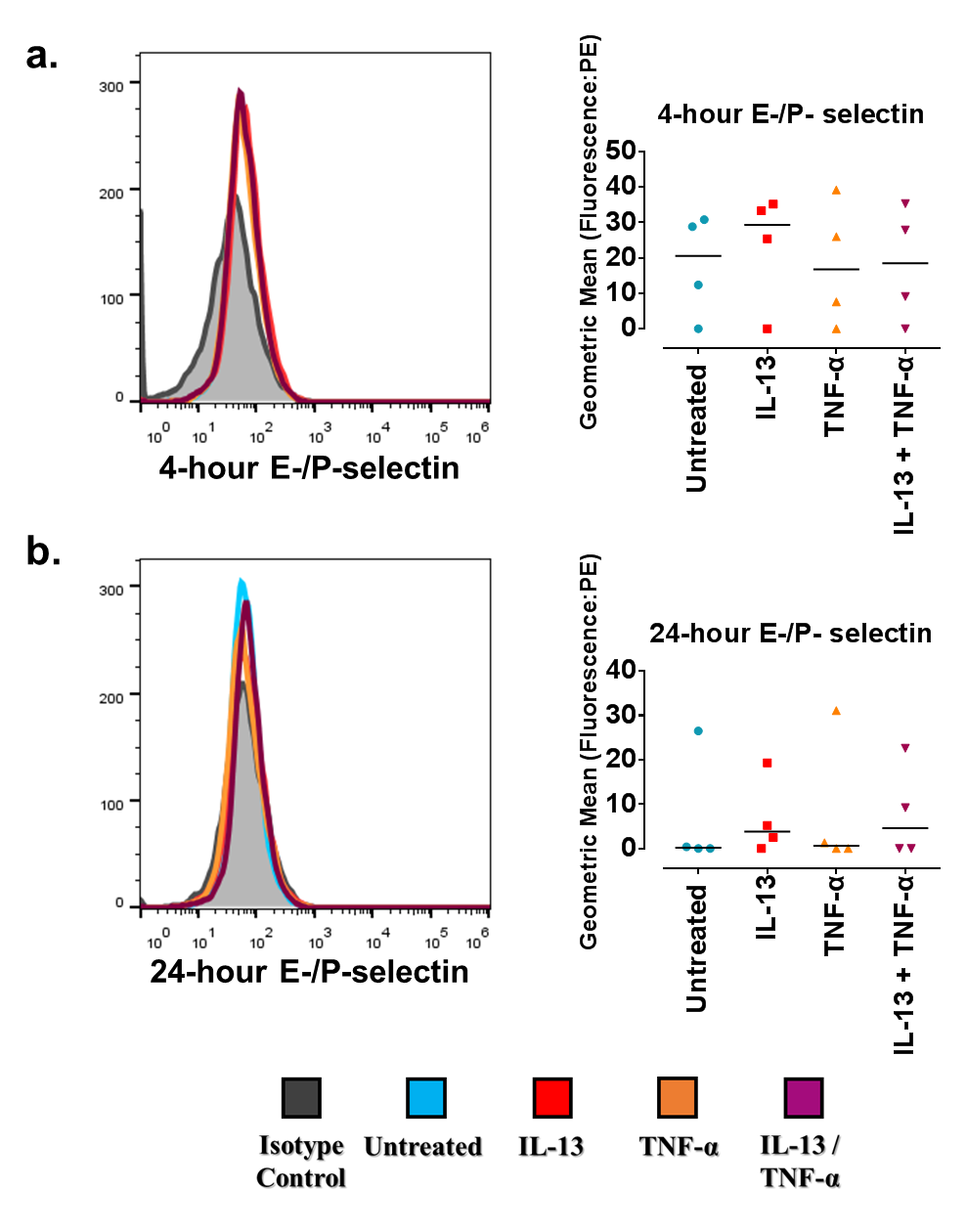
**

Supplementary Figure 3


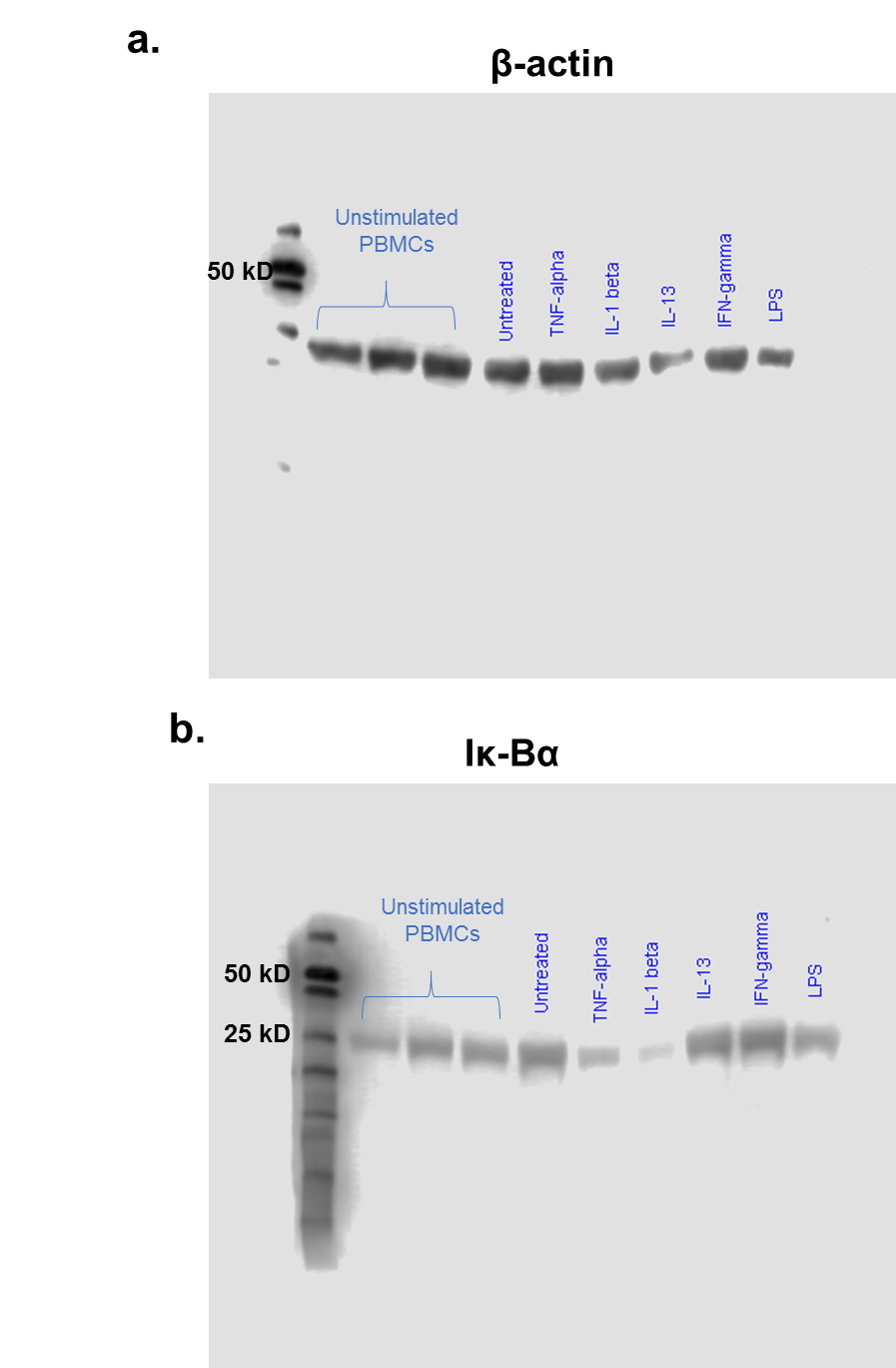


**Supplementary Figure 4
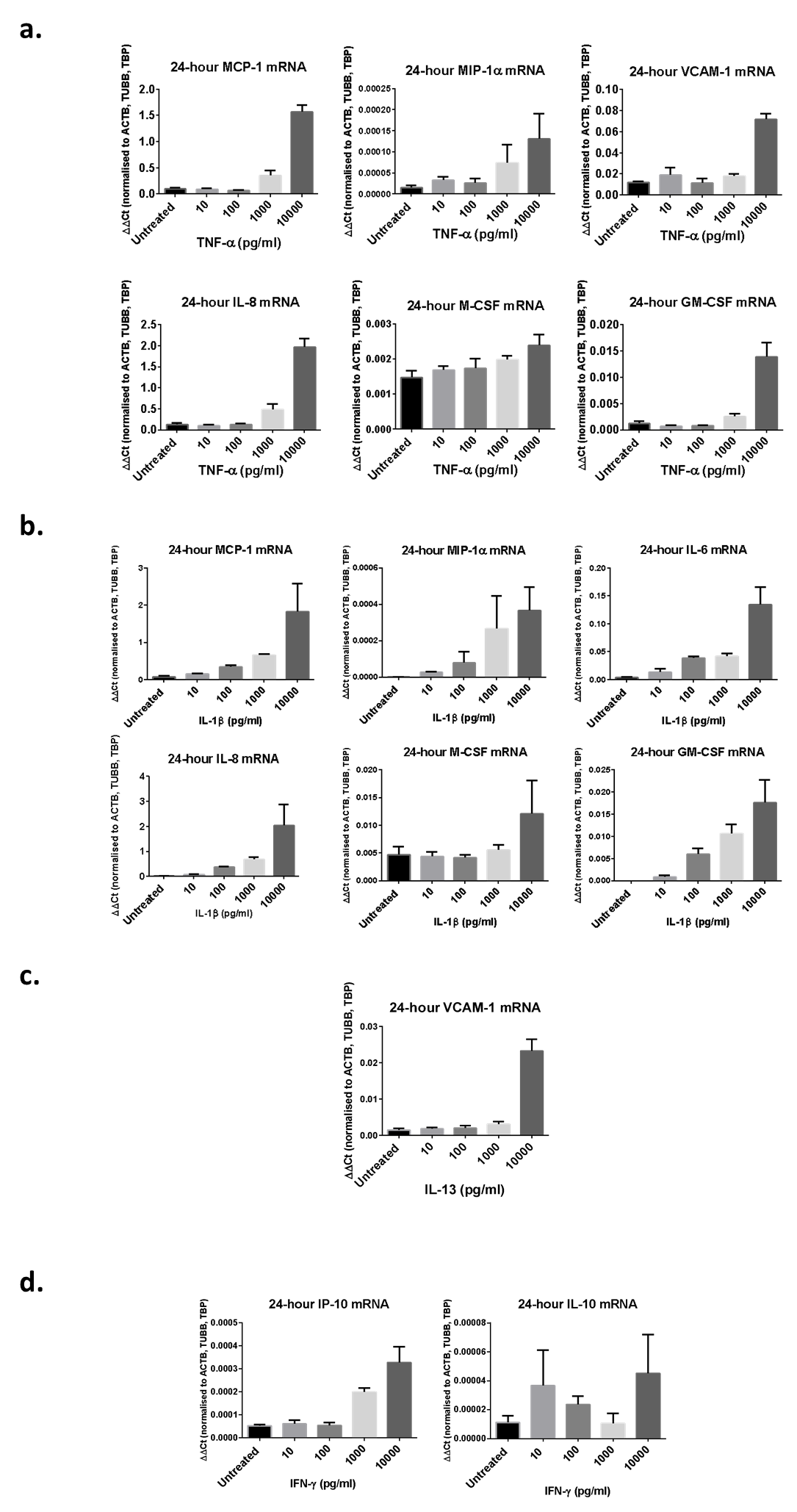
**
